# Interacting with fallible AI: is distrust helpful when receiving AI misclassifications?

**DOI:** 10.3389/fpsyg.2025.1574809

**Published:** 2025-05-27

**Authors:** Tobias M. Peters, Ingrid Scharlau

**Affiliations:** Department of Psychology, Faculty of Arts and Humanities, Paderborn University, Paderborn, Germany

**Keywords:** trust in AI, trust, distrust, human-AI interaction, Signal Detection Theory, Bayesian parameter estimation, image classification

## Abstract

Due to the application of artificial intelligence (AI) in high-risk domains such as law and medicine, trustworthy AI and trust in AI are increasingly relevant to science and the public. A typical conception, for example, in the context of medical diagnosis, is that a knowledgeable user receives AI-generated classifications as advice. Research to improve such interactions often aims to foster users' trust, which, in turn, should improve combined human-AI performance. Given that AI models can err, we argue that the possibility of critically reviewing and thus distrusting an AI decision is an equally interesting target for research. We created two image classification scenarios in which participants received mock-up AI advice. The quality of the advice decreases during a phase of the experiment. We studied task performance, as well as participants' trust and distrust, and tested whether an instruction to remain skeptical and to review each piece of advice led to better performance compared to a neutral condition. Our results indicate that this instruction does not improve but rather worsens the participants' performance. Repeated single-item self-reports of trust and distrust indicate a decrease in trust and an increase in distrust following the decline in AI's classification quality, with no difference between the two instructions. Furthermore, through a Bayesian Signal Detection Theory analysis, we provide a procedure to assess appropriate reliance in detail by quantifying whether the issues of under- and over-reliance have been mitigated. We discuss the implications of our results for the usage of disclaimers before interacting with AI, as prominently used in current LLM-based chatbots, and for trust and distrust research.

## 1 Introduction

Given the widespread use and adoption of applications based on artificial intelligence (AI), trustworthy AI and trust in AI have become areas of scientific and societal interest and relevance. The possibility that AI affects decisions in high-stakes areas such as medicine, law, or finance has especially accelerated this interest. Indicative of this, research on trust in AI has increased rapidly across disciplines in the last few years (Benk et al., 2024), and numerous guidelines and initial legislation provide recommendations, checklists, or legal requirements that aim to ensure trustworthy AI (Thiebes et al., 2021; Hohma and Lütge, 2023; Regulation (EU) 2024/1689, 2024). In high-risk use cases, decisions should not be made solely by AI. The dominant conceptions are that humans team up with AI (Capel and Brereton, 2023), achieve complementary performance (Guo et al., 2024), or are advised by AI (Dhanorkar et al., 2021; Agarwal et al., 2023). In a similar vein, adopting the EU AI act would require human oversight for those applications categorized as high-risk (Regulation (EU) 2024/1689, 2024, article 14).

Ultimately, trustworthy AI should allow people to trust AI in a way that maximizes the benefits of AI advancements while preventing or at least mitigating its risks (Thiebes et al., 2021). “People” in this context may refer to a single user, stakeholders, or the broader public; correspondingly, the precise scope of the objective of trustworthy AI differs. In addition to the differing scopes, the meaning of trust in this context is often not explicitly defined. As Bareis (2024) highlights, the European AI Act, which is currently regarded as the most influential framework for trustworthy AI, fails to define trust even once. This lack of an explicit definition can also be observed in the current research literature (Benk et al., 2024; Ferrario and Loi, 2022), which can lead to a conflation of defined concepts of trust and common-sense reasoning about trust.

This is problematic because trust is a complex concept, and failing to define it can result in misunderstandings, misuse, or neglect of its inherent qualities. Moreover, and central to this paper, we find it important to consider distrust in this context as well. Ideally, people should trust AI when it is correct and distrust it when it is incorrect. This distinction and focus on distrust may seem superfluous when considering trust and distrust as opposing ends of one continuum. However, research increasingly suggests that the two are better considered as related yet separate dimensions (Lewicki et al., 1998; Kohn et al., 2021; Scharowski et al., 2024). This two-dimensional model, prominently proposed in organizational science by Lewicki et al. (1998), has since gained relevance (Vaske, 2016), although it has received little recognition in AI research (though see Kohn et al., 2021; Colville and Ostern, 2024; Scharowski et al., 2024).

Technological advancements preceding the recent developments in AI have already resulted in increasingly complex interactions between humans and technology that involve both uncertainty and risk. These characteristics of interactions, uncertainty and risk, are also common in interpersonal interactions (Mayer et al., 1995). Interactions that can be highly complex, and trust serves as a mechanism to reduce this complexity (Luhmann, 2009). This commonality led to the consideration of interpersonal trust research in the context of automation. Prominently, the foundational work on trust by Mayer et al. (1995), which clarified the distinctions between trust, its antecedents, outcomes, and the role of risk, has been adopted in the context of trust in automation (Lee and See, 2004; Hoff and Bashir, 2015), subsequently influencing research on trust in AI (Benk et al., 2024).

In current research on trust in AI, it is common to focus on appropriate trust (or comparable concepts such as warranted or calibrated trust; Mehrotra et al., 2024). The inclusion of adjectives such as appropriate, warranted, or calibrated acknowledges that AI is fallible and that there are situations where it is wrong to trust and right to distrust. However, what exactly constitutes appropriate, warranted, or calibrated trust remains largely descriptive and conceptually vague. In their systematic review, Mehrotra et al. (2024) report that 75% of articles on appropriate trust in AI did not define appropriate trust or related concepts.

These concepts share the idea that ideally, neither under-reliance nor over-reliance should occur; that is, one should not rely on correct AI advice nor on incorrect AI advice, respectively. Numerous studies on explainable AI (XAI; e.g., Samek et al., 2021; Mohseni et al., 2021) are currently investigating various methods to improve interactions with AI by incorporating explainability methods. Typically, the use of explainability methods is assumed to facilitate trust, which has been coined as the explainability-trust hypothesis (Kastner et al., 2021). By scrutinizing this assumption, we argue that it neglects the fact that explainability methods can serve two functions (Peters and Visser, 2023). They can help not only to identify reasons to trust but also to identify reasons to distrust. This aligns with the observation by Kastner et al. (2021) that there are more careful formulations of the explainability-trust hypothesis, which do not aim to increase trust but rather to promote appropriate trust instead (Kastner et al., 2021).

Such formulations of the explainability-trust hypothesis acknowledge the second function—identifying reasons for distrust—but the majority of user studies still primarily assess trust. Only 14% of studies on appropriate trust consider distrust (Mehrotra et al., 2024). The problem of over-reliance on AI (e.g., Spatharioti et al., 2023; Vasconcelos et al., 2023) illustrates that it may be beneficial for human-AI interactions to foster distrust by using the second function of explainability effectively. Furthermore, we see merit in focusing on trust and distrust as two related yet separate dimensions because this allows for the co-existence of the two, which may resolve a conceptual shortcoming of the one-dimensional approach to conceptualizing appropriate trust. For instance, a foundational concept of trust (Mayer et al., 1995) defines trust as the willingness to rely, irrespective of the ability to monitor or control. The majority of descriptions of appropriate trust revolve around the alignment between the perceived and the actual capabilities of an AI (Mehrotra et al., 2024).

Evaluating this alignment process solely by trust may overlook or only indirectly assess important aspects of it. Trust, as the willingness to rely, may be present even if a person is unable to perceive AI's capabilities or specifically because a person can accurately perceive them. Whether the former or latter holds true cannot be distinguished by the amount of trust measured. However, measuring whether some distrust is present may provide a way to make this distinction because, in the two-dimensional approach, aspects like skepticism, wariness, and vigilance are characteristics of distrust (Lewicki et al., 1998; Cho, 2006).

Of course, in many cases, high trust is associated with low distrust, which the two-dimensional models also assume (Lewicki et al., 1998). However, the specific qualities of trust and distrust differ, and trust and distrust can co-exist in certain situations that align well with the description of appropriate trust. For instance, Lewicki et al. (1998) describe that in situations where both trust and distrust are high, individuals pursue opportunities while continuously monitoring risks. Similarly, for AI applications, this would mean that a user utilizes AI while double-checking certain information (Scharowski et al., 2024).

Therefore, we find it helpful to theoretically base the notion of appropriate trust on the two-dimensional conceptualization of trust and distrust. Following Mayer et al. (1995); Lee and See (2004); Hoff and Bashir (2015), we define trust in AI as the willingness to rely on AI based on the expectation that this will help achieve an individual's goal in a situation characterized by risk and uncertainty. Similar to Lewicki et al. (1998), we define distrust in the AI context in reciprocal terms to trust, i.e., as the hesitance to rely, while at a detailed level, its characteristics are different from those of trust, as described above.

### 1.1 Methodological background

In summary, we find both from a conceptual perspective and from an application perspective that there is reason to focus on distrust in the context of human-AI interaction. Distrust may be an important ingredient for appropriate trust. XAI may foster it, and only assessing trust and distrust one-dimensionally falls short of evaluating this properly. To approach this empirically, scenarios with fallible AI and means that aim to improve human-AI interaction by mitigating over-reliance need to be investigated. As a scenario, we chose AI-based image classifications. They are increasingly adopted in different areas and contexts. Their applications can range, for example, from classifying everyday objects (Redmon, 2016), plants (Mäder et al., 2021), or mushrooms (Leichtmann et al., 2023) to melanoma (Brinker et al., 2019) or mammograms (Calisto et al., 2021). Therefore, we focus on AI-interactions in which users encounter false classifications to investigate how distrust may be helpful to prevent over-reliance and thereby may improve the combined human-AI performance. In doing so, this study investigates the following research questions:

- RQ1: Is it helpful to foster a user's distrust before interacting with erroneous AI?
- RQ2: Do people notice worsening AI advice, and how does it affect their trust and distrust?

To this end, we study task performance, trust, and distrust in an image classification experiment where participants are supported by a mock-up AI. The quality of the AI's classifications decreases during a phase of the experiments. To investigate the first research question, we test whether an instruction to remain skeptical and critically review this AI advice (distrust instruction) leads to better decision performance compared to a control condition with neutral instructions (information-only instruction). Since the ground truth of the AI classification in this mock-up scenario is known, we can determine if any potential improvement arises from mitigating over-reliance.

We approach the second research question by analyzing whether, and if so, how the decreased quality of the AI's classifications affects participants' self-reported trust and distrust. The self-report is repeatedly measured. The actual reliance behavior is assessed via the AI-advised categorization made by the participants. The repeated measurement of trust and distrust is chosen due to the dynamic nature of trust (Luhmann, 2009; Hoff and Bashir, 2015). Self-reported trust and distrust, as well as reliance, are both assessed due to the potential discrepancy between trusting intentions and trusting behavior (Papenmeier et al., 2019, 2022; Wang and Yin, 2023). Although self-reported (dis)trust and reliance behaviors are conceptually distinct, we included a self-report measure of reliance as a control variable.

The study does not include actual AI-generated classification (a procedure widely applied and described as the Wizard of Oz Design; for example, see Lai et al., 2021). The supposed AI classifications are constructed (see Section 2) to allow for a known ground truth and to easily distinguish between cases in which trust or distrust toward AI classifications would be appropriate. Furthermore, the study includes only the classification without any descriptors or XAI methods. This enables us to analyze the classification's correctness without any potentially influencing factors (e.g., uncertainty estimates). During the second phase of the study, the AI advice became substantially less accurate. If participants relied on this advice, their performance was expected to decline accordingly.

If the distrust instruction helps mitigate over-reliance, participants who receive it should show a smaller performance drop when given incorrect advice. Thus, as *Hypothesis 1*, we expect better performance for the participants in the distrust condition than for those in the information-only condition. Furthermore, we expect participants to notice the worsening of advice. Therefore, as *Hypothesis 2a*, we expect a decrease in self-reported trust and, as *Hypothesis 2b*, an increase in self-reported distrust due to the second phase of the study. Due to the prolonged exposure to, and higher frequency of errors in, the second phase, we expect participants to be more aware of wrong advice, which leads to an improved rejection of wrong advice and, consequently, an increase in performance. Therefore, as *Hypothesis 3*, we expect higher performance after the second phase than before it.

## 2 Materials and methods

We conducted a study that was divided into two sessions. In Session 1, all participants categorized the image material without AI advice. In the main experiment of the study (Session 2), the participants had to categorize the same image material but received mock-up AI advice. The main experiment consisted of the between-subject factor Condition and the within-subject factor Advice Correctness. The participants were randomly assigned to either the distrust condition or the information-only condition. Depending on the condition, they received different instructions about their interaction with the mock-up AI. The within-subject factor Advice Correctness varied depending on the phase of the study, which includes the pre-error, error, and post-error phases.

We assessed the participants' categorizations both without AI advice (Session 1) and with AI advice (Session 2). This allowed us to take the participants' individual performance differences into account. Both types of categorizations, combined with Advice Correctness, enabled us to assess whether participants improved due to correct AI advice and worsened due to incorrect advice, which served as detailed indicators of appropriate reliance. For this analysis of the behavioral data, we used Signal Detection Theory (SDT), as explained in Section 2.5.1. In line with previous studies on reliance (e.g., Zhang et al., 2020, 2022; Naiseh et al., 2023), we also report acceptance rates and switch percentages as another measure of reliance and compare them between conditions. We calculated the acceptance rate using the frequency of how often the participants' decisions in Session 2 were the same as the AI advice they received. We derived the switch percentage from the frequency of how often the participants decided as advised by the AI, even though they decided differently in Session 1.

Furthermore, we repeatedly measured self-reported trust and distrust. Self-reports were measured via single items on a 7-point Likert scale (“How much do you ‘trust'/‘distrust' AI advice?,” 1 = “not at all,” 7 = “completely”). In the same manner, we also asked how much they used the advice. This measure served as a control variable, as mentioned above. In each trial of both sessions, the participants rated how certain they were about their decision on a 7-point Likert scale ranging from very uncertain to very certain. The certainty was assessed for purposes outside the scope of this paper and is thus not included in the present analysis.

To improve generalizability and compare different settings, we developed two image classification scenarios (geometric forms and real or fake images; see below). We ran two versions of the same experiment for each of these scenarios, once in a laboratory and once in an online environment. Therefore, we conducted a total of four experiments, each divided into two sessions. In Session 1, we assessed how well participants performed without AI advice in order to consider their individual performance when analyzing the results of Session 2. All experiments were approved by the ethics committee of Paderborn University. The experiments were created with jsPsych (Version 4.4.0) and hosted on a Jatos server.^1^

### 2.1 Participants

For the laboratory experiments, we recruited German-speaking students enrolled at Paderborn University. For the geometric forms scenario, people with visual color deficiencies were excluded from participation. Furthermore, we excluded current or former computer science students, as they are less likely to be convinced by the cover story of the experiments (see below). Participation was compensated either with 10€ per hour or with course credit. In order to create some risk for the participants, the three best participants in the AI-supported classification task were rewarded with a bonus payment as recommended by Miller (2022). The bonus was 40€.

Before the first session, all participants provided their informed consent to the terms of the experiment and data processing, and they were informed about the potential bonus payment. All participants in the laboratory experiments (*N* = 61) had normal or corrected-to-normal vision and were between 18 and 39 years old (*M* = 21.69, *SD* = 3.48). 78.68 % of the participants identified as female. For the online experiments, we recruited participants via Prolific.^2^ We allowed only participants who reported German as their first language. Participation was rewarded with 9£ per hour, and the three best-performing participants also received a monetary bonus. The online participants (*N* = 70) were between 21 and 74 years old (*M* = 34.5, *SD* = 10.64). 36.11 % of the participants identified as female.

### 2.2 Stimulus material

We created two classification scenarios: the Forms Scenario and the Real or Fake (RoF) Scenario. The Forms Scenario involves abstract figures that the participants classify based on unknown rules, while the RoF Scenario includes pictures of faces or everyday objects that are to be classified as real or fake. In the Forms scenario, we present stimuli that are new to the participants. The real images of the RoF material are stimuli which the participants encounter frequently and with which they are thus highly familiar. Furthermore, the Forms material is highly standardized and visually less complex than the RoF material, and each stimulus is easily comparable in terms of classification difficulty. The RoF material is visually highly complex but less standardized and also more difficult to compare in terms of classification difficulty. These differences are not relevant to the present study. However, they would be relevant for investigating and modeling the participant's perception of the stimuli in more detail.

In the RoF Scenario, images were classified as either real (actual photographs) or fake (AI-generated images). Two types of real or fake images were used: faces and everyday objects.^3^ We selected two subsets to create a stimulus set that is neither too difficult nor too easy. Pilot studies indicated that the face subset was very challenging for some participants, while the object subset was relatively easy for others to classify. Combining the two subsets yielded a satisfactory level of difficulty. Inspired by *whichfaceisreal.com*, the fake images of faces were sourced from *thispersondoesnotexist.com*.^4^ The real images of faces were obtained from the flickr-faceHQ-database (Karras et al., 2019). As a secondary subset, images depicting objects, landscapes, or animals were used. The images of fake objects were created using Bing Image Creator, while the real objects were sourced from the THINGS database (Hebart et al., 2019).

For the Forms Scenario, we generated two-dimensional geometric forms that can be categorized as belonging to one of two categories. The categorization rules are based on the form's color, its width-to-height ratio, and the type of curvature (convex vs. concave). The forms are categorized as Type A if they are either:

- blue, have a concave line, and are wider than high, or
- red, have a concave line and are higher than wide.

They are categorized as Type B if they are either:

- blue, have a convex line, and are higher than wide or
- red, have concave lines and are wider than high.

The forms were created in Python with *matplotlib*.^5^ All forms have five points, five straight lines, and one curved line. 50% of the forms are clearly higher or wider (ratio between height and width above 1.5), and 50% are wider or higher (ratio between height and width between 1.05 and 1.5). In a pilot study, we tested how well participants could learn the categorization from the information provided to them (see Section Procedure). Some participants solved the task with very few mistakes. Some performances suggested a partial understanding of the categorization rules, while a few participants were unable to identify the rules and stayed at or near chance level. This variance in task performance indicated adequate task difficulty.

### 2.3 Procedure—Laboratory experiments

All participants included in the analysis took part in both sessions. The sessions had to be at least one day but <10 days apart. Both sessions were conducted in a dimly lit laboratory with one indirect light source behind the PC screen. A 24” screen with a resolution of 1,920 × 1,080px was used in all experiments. The images were presented against a white background (RGB: 255, 255, 255). Throughout both sessions, short breaks were offered at regular intervals.

#### 2.3.1 Session 1

Depending on the scenario, the participants were informed that they would be presented with 2D geometric forms or real or fake images, which they had to categorize as one of two categories: Type A or Type B, or real or fake, respectively. In the Forms Scenario, the participants received more extensive information about the material and were also given the chance to familiarize themselves with it and complete practice trials.

The participants were informed that the color, the curved line, and the width-to-height ratio of the forms can be decisive in their categorization. They were told that all forms can be classified into one of the two categories. During the familiarization part, the participants reviewed four examples of correct classifications for both types and a ninth form placed in the middle. They needed to determine whether the middle form was Type A or Type B. They were instructed to try to learn the categories by heart. In the practice part, the participants saw a single form and had to judge whether the form was Type A or B.

The familiarization and practice parts were each presented twice, which the participants were informed of in advance. The familiarization consisted of 8 blocks of 4 trials. For each block, the presented examples changed. It was ensured that two blue and two red examples were always presented and that their ordering was (pseudo-)randomized. After that, the first short practice part, consisting of 10 trials, followed. Then, the familiarization part was repeated with different images, followed by a longer test practice with 20 trials. In both parts, the participants received auditory feedback based on the correctness of their decisions.

**Figure 1:**
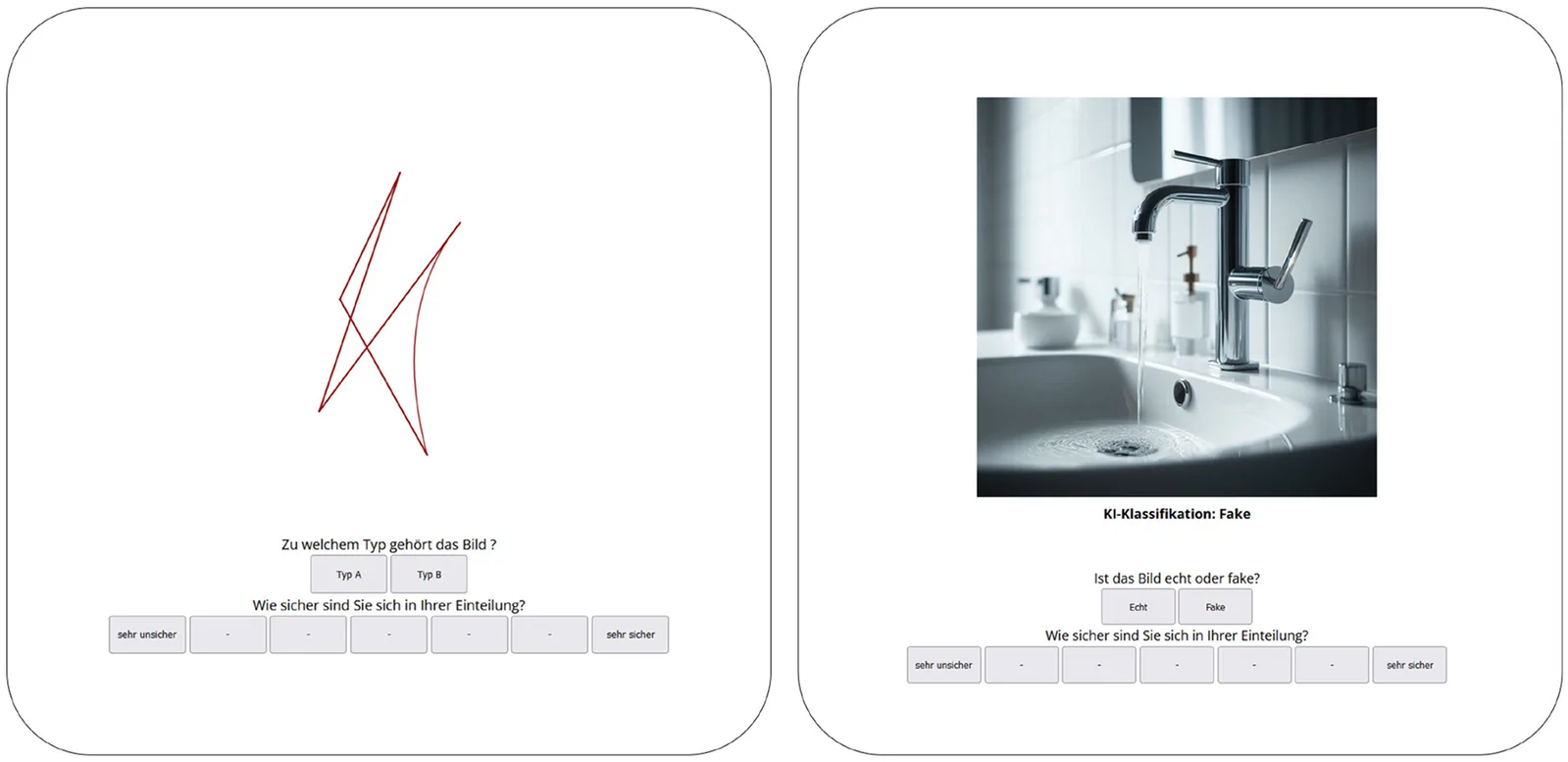
Study setup of Session 1 with Forms material (**left**) and study setup of Session 2 with RoF material (**right**). For illustration, we show an example of the Forms material for the Session 1 setup and an example of the RoF material for the Session 2 setup. However, for the participants, only one type of material was presented in both sessions.

After the practice parts, the procedure was again identical for both scenarios. The participants had to categorize single forms or images as either Type A or B, or as real or fake, respectively (see Figure 1 left). All participants saw the same stimuli. The order of stimuli was randomized for each participant. The main experiment comprised 8 blocks of 27 trials (i.e., 216 stimuli). In the Forms Scenario, unbeknownst to the participants, 54 blue and 54 red forms of each type were used. In the RoF Scenario, half of the images were faces and half were objects, with 50% being real and 50% being fake images. At the end of Session 1, participants could answer two open-ended questions that asked how they determined the category to which the stimuli belonged.

#### 2.3.2 Session 2

Session 2 followed the same general procedure as Session 1. The main difference was that participants were verbally informed that they would receive AI support. They were informed about the capabilities of the supposed AI depending on the condition (distrust or information only; see below) they were in. This information was provided in advance to ensure that participants adhered to the instructions. At the beginning of the experiment, participants were reminded of the potential bonus payment and informed that it was based on their performance during the AI-supported session. The task instructions were displayed on screen.

In the Forms Scenario, participants were informed that they could refresh their memory with a few practice trials. They completed three blocks comprising four training trials and 20 test trials. If a participant answered fewer than 75 percent of the test trials correctly, both the training and test trials were repeated once with different stimuli. Afterward, all participants proceeded to the experimental trials, which again followed the same procedure in both scenarios.

The participants were informed that they would receive AI support moving forward and were provided with information about the AI's benefits and potential issues. Participants in the information-only condition were informed that a validation test revealed that the current AI model was very good at the task and, while some errors may still occur, the AI generally performs well on the task and that they should make use of its classifications.

Participants in the distrust condition received the same information but in a different order. They were informed that the errors that may occur can be very obvious to humans, and that even for errors which are not obvious, previous studies have shown that human intuition provides a useful indicator for potential errors. They were advised to make use of the AI classifications but to remain cautious and decide for themselves when to rely on it (The exact instructions and their translation can be found in the Supplementary material).

For the AI-supported task, the setup of Session 1 was simply extended by the mock-up AI advice that indicated AI classification: Type A or Type B, or Real or Fake (see Figure 1 right). Unbeknownst to the participants, the accuracy of the supposed AI advice was determined by Bernoulli sampling with a likelihood of 0.9 in the pre- and post-error trials. During the error trials, this likelihood decreased block-wise (0.75, 0.6, 0.45). Each block consisted of 24 trials, including three blocks of pre-error trials, three blocks of error trials with decreasing accuracy, and three blocks of post-error trials. In total, 216 forms or images were evaluated with AI support.

After every two blocks and after the final block, participants answered three questions. These time points were chosen so the queries did not always coincide with changes in the likelihood of receiving correct advice. Participants rated how much they used the AI classification, how much they trusted it, and how much they distrusted it on a 7-point Likert scale ranging from very little to very much.

At the end of Session 2, the participants were asked again how they determined to which category the stimuli belonged. Furthermore, they were asked how familiar they were with AI-supported image classification and how familiar they were with AI-based applications in general. Afterward, participants were debriefed about the purpose of the experiment.

### 2.4 Procedure—Online experiments

The procedure for the online experiments mostly followed the steps described above. The participants were instructed that they could not use mobile devices for participation. At the beginning of each session, we recorded the participants' browser type, width, and height to ensure adequate conditions for participation. If the minimum specifications (Forms Scenario: 1,600 × 920px; RoF Scenario: 1,024 × 768px) were not met, the participants were asked to sufficiently adjust their browsers. If this was not possible, they were excluded from participation.

The online participants received the same written instructions as the laboratory participants and were required to summarize them to ensure their understanding.

### 2.5 Statistical analysis

The results were analyzed using RStudio (R version 4.4.0) and Jupyter Notebook (Python version 3.10.5). Within the Jupyter Notebook framework, we used the *pymc4* library for the Bayesian SDT analysis. The Bayesian analysis of the contingency tables for the acceptance rates and switch percentages, which was added for reviews, and the Bayes factors were calculated with JASP (version 0.19.3, JASP Team, 2025). The remaining statistical analyses were carried out in R. For the Bayesian linear mixed regressions, we used the *brms* package (Bürkner, 2017).

#### 2.5.1 Signal Detection Theory

For the SDT analysis, we adapted the Bayesian modeling approach described in Lee (2008); Lee and Wagenmakers (2014). Unlike the typical method, which calculates sensitivity and bias parameters as single values, this approach enables us to quantify the uncertainty associated with these values. By design, our data include more trials where the AI advice is correct than incorrect. This unequal count of observations results in differing certainty about the SDT parameters. The Bayesian framework inherently captures this aspect, whereas classical approaches do not (Lee, 2008).


(1) $$\begin{array}{l} \begin{array}{cc} h_{i} & =\Phi \left(\frac{1}{2}d_{i}-c_{i}\right)\\ f_{i} & =\Phi \left(-\frac{1}{2}d_{i}-c_{i}\right) \end{array} \end{array}$$

**Figure 2:**
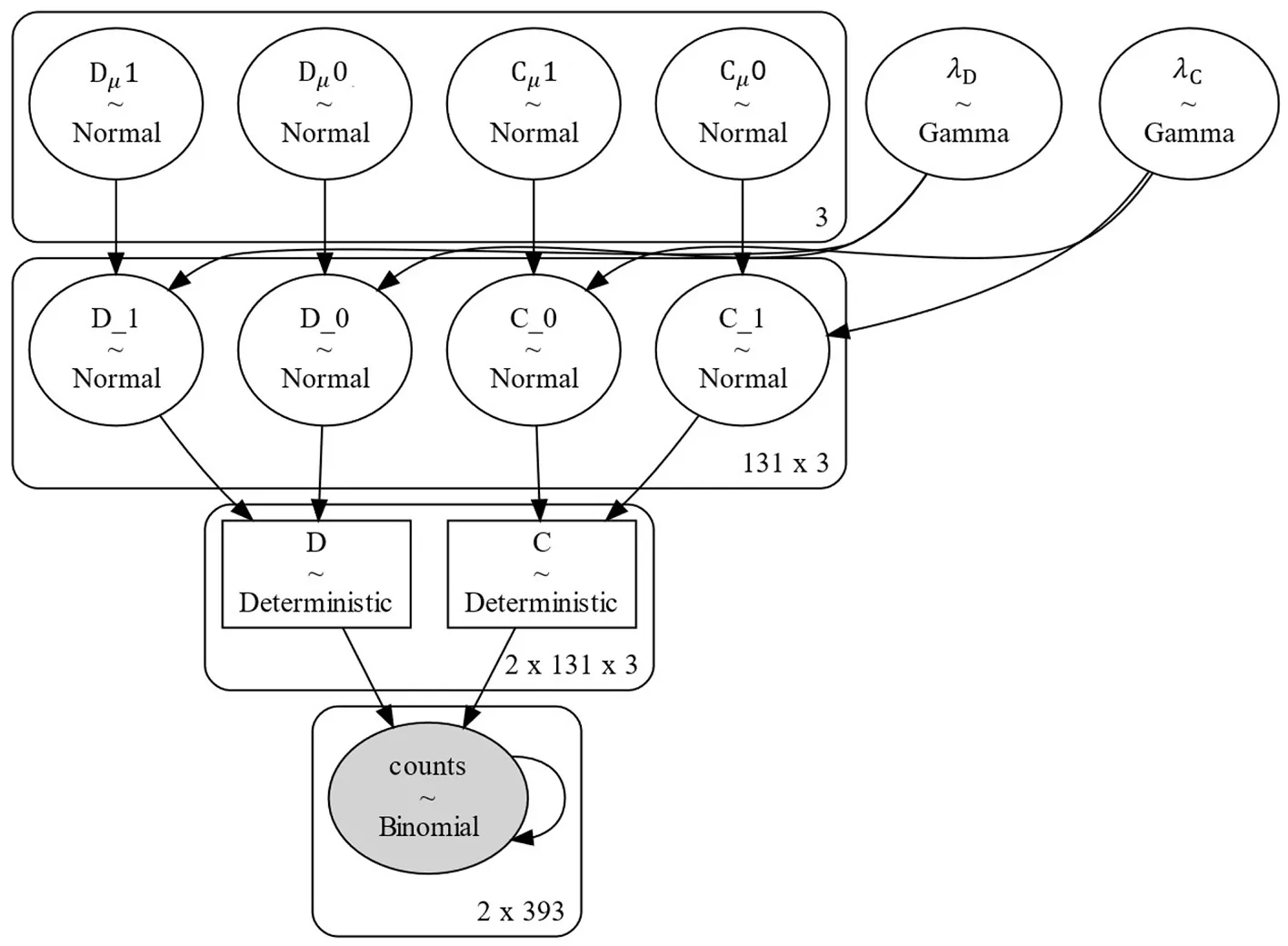
Hierarchical Bayesian SDT model structure. The indices of *D*_μ_ and *C*_μ_ represent the two conditions. The “x 3” stands for the shape of the priors, which in this visualization is based upon the three phases of the experiment.

We used a hierarchical Bayesian model (see Figure 2) in which for each condition, the *d*′ and *c* values of each participant are drawn from the overarching normal distributions *D*_μ_ and *C*_μ_, respectively. As priors for the precision of each participant's values, we chose the Gamma distributions λ_*D*_ and λ_*C*_. The *d*′ and *c* values are reparametrized into the hit and false alarm rates *h*_*i*_ and *f*_*i*_, as detailed in Equation 1, with Φ representing the standard cumulative Gaussian function. To avoid problems with encountering frequencies of 0 and following (Hautus et al., 2021), a log-linear correction was applied that adds 0.5 to all frequencies. The priors we used are as follows:


(2) $$\begin{array}{l} \begin{array}{cc} D_{\mu } & \sim Gaussian\left(0,1.9\right)\\ C_{\mu } & \sim Gaussian\left(0,.95\right)\\ \lambda_{D}|\lambda_{C} & \sim Gamma\left(.001,.001\right)\\ D_{i} & \sim Gaussian\left(D_{\mu },\lambda_{D}\right)\\ C_{i} & \sim Gaussian\left(C_{\mu },\lambda_{C}\right) \end{array} \end{array}$$

The *d*′ and *c* priors (see Equation 2) were chosen based on the theoretically possible values for these parameters. At most, 216 trials are included in the parameter estimations, and thus a maximum of 108 hits and false alarms could occur. Given the applied log-linear correction, the hit and false alarm rates can range between $\frac{0.5}{109}$ and $\frac{108.5}{109}$. Therefore, *d*′ values between −5.21 and 5.21 and *c* values between −2.61 and 2.61 are theoretically possible. Thus, we ensured that the theoretically possible values are within 3*SDs* of the Gaussian priors. For each session, we ran individual models with four chains with 10,000 samples, while the first 2,000 were used as tuning samples and were not included in the posteriors.

From the *d*′ estimates of the two sessions, we calculated the *d*′ difference between Session 2 and Session 1 for each participant. Thereby, we quantified individual improvement or worsening based on AI advice they received in Session 2. This was done twice: once using all trials from Session 2 and once by splitting the trials from Session 2 according to Advice Correctness. The former is used to quantify performance changes due to AI advice in general to test *Hypothesis 1*. The latter allows us to inspect these changes in more detail and is therefore referred to as the detailed *d*′ difference. A positive *d*′ difference indicates that the participant benefitted from the AI advice, a negative difference indicates that their performance worsened due to the advice, and a difference of zero signals no change due to the advice.

The detailed *d*′ difference provides two values for each participant: the difference when the advice was correct and the difference when the advice was incorrect. This enables us to quantify whether the participants improved by generally relying on the AI advice or by appropriately relying on it. In the case of general reliance, the detailed *d*′ differences would be positive when the advice was correct and negative when it was incorrect. The case of appropriate reliance is indicated by a positive detailed *d*′ difference when the advice is correct and a detailed *d*′ difference close to zero when the advice is incorrect.

## 3 Results

### 3.1 Behavioral results

In Table 1, we report the descriptive statistics for the Bayesian SDT performance estimate *d*′ from both sessions, along with the detailed *d*′ differences. From this, we observe an improvement in the participants' performance in Session 2 compared to Session 1. On average the *d*′ values were higher when the advice was correct than when the advice was incorrect. This indicates that the participants' decisions were influenced by the advice they received. Furthermore, the acceptance rates and switch percentages of the participants were higher when the advice was correct than when it was not (see Table 2).

**Table 1:** Descriptive statistics for *d*′ values of the Session 1 & 2.

	**n**	**M**	**SD**	**Median**	**Min**	**Max**
Session 1	131	0.95	0.83	0.96	-0.30	3.86
Session 2	131	1.83	0.90	1.71	0.15	4.46
Session 2 - advice correct	131	2.46	1.09	2.24	0.14	4.85
Session 2 - advice incorrect	131	0.28	1.56	0.43	–3.33	3.51

Lower half: *d*′ values of Session 2 split by Advice Correctness.

**Table 2:** Contingency table for the acceptance rate and switch percentages split by Advice Correctness.

**Advice correctness**	**Advice accepted**		**Switched to advice**	
	**No**	**Yes**	**No**	**Yes**
Incorrect	3, 147 (54.81%)	2, 595 (45.19%)	1, 325 (34.55%)	2, 510 (65.45%)
Correct	3, 266 (14.48%)	19, 288 (85.52%)	5, 627 (74.19%)	1, 958 (25.81%)
Total	6, 413 (22.66%)	21, 883 (77.34%)	6, 952 (60.87%)	4, 468 (39.12%)

Difference by Advice Correctness for acceptance rate: *BF*_10_>10, 000.

Difference by Advice Correctness for switch percentages: *BF*_10_>10, 000.

**Figure 3:**
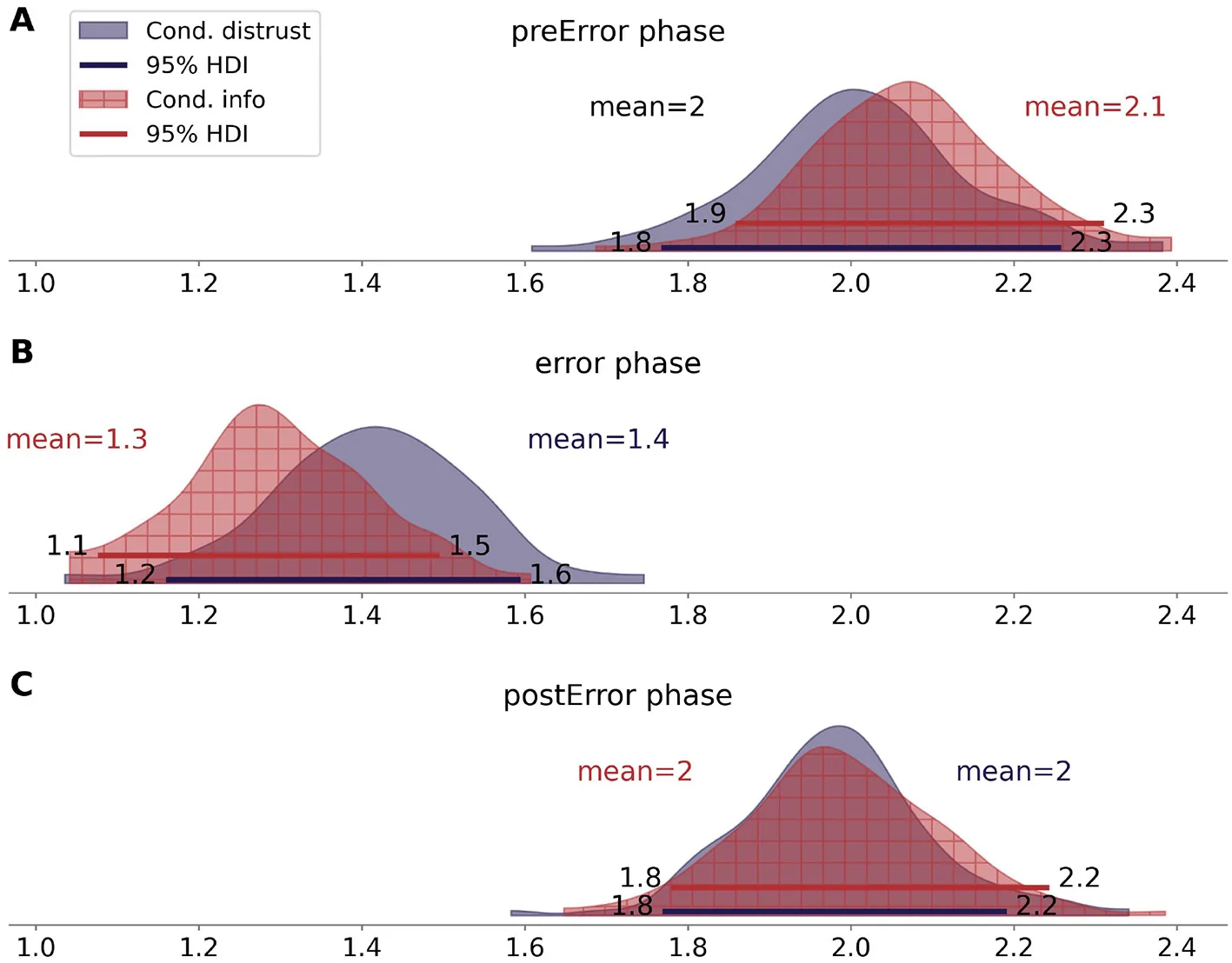
Posterior density distributions of *d*′ split by Condition and phase (**A**: pre-error phase, **B**: error phase, **C**: post-error phase).

Figure 3 shows the *d*′ values from Session 2 and their variation across phases for each condition. In the pre-error phase, the *d*′ estimate in the information-only condition centers at 2.0 [1.8, 2.3] and at 2.0 [1.8, 2.2] in the distrust condition. In the error phase, both estimates drop to mean values of 1.3 [1.1, 1.57] and 1.4 [1.2, 1.6] in the information-only and the distrust condition, respectively. In the post-error phase, the mean *d*′ values are again at 2 [1.7, 2.2] in the information-only condition and 2 [1.7, 2.2] in the distrust condition. These results show the expected drop in performance in the second, i.e., the error phase of the study for both conditions. However, these results do not support *Hypothesis 3*; that is, they do not suggest that the performance is higher after the second phase than before it.

**Figure 4:**
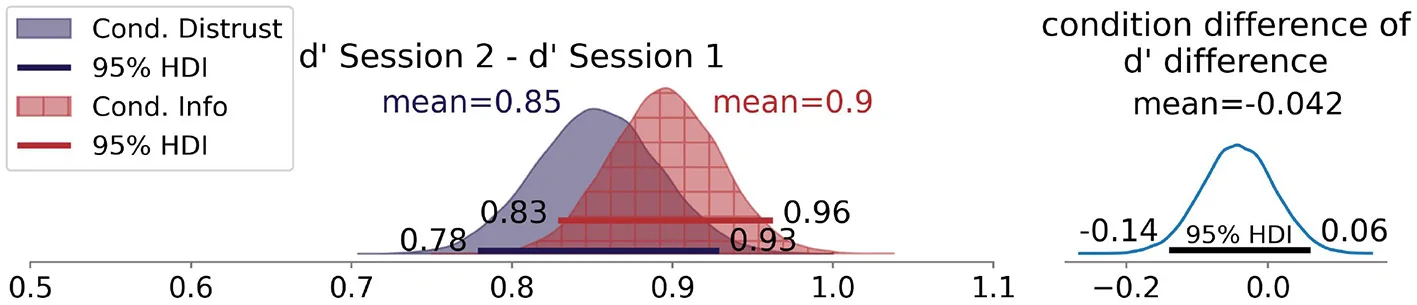
Posterior density distributions of the *d*′ difference between Session 2 and Session 1 split by Condition.

To test whether the AI-advised performance of the participants improved after receiving the distrust instruction, we compared the *d*′ differences between conditions. Contrary to our expectations formulated in *Hypothesis 1*, Figure 4 shows that the improvement in Session 2 in the distrust condition was slightly lower (*M* = 0.85, [0.78, 0.93]) than the improvement in the information-only condition (*M* = 0.89, [0.82, 0.95]).

**Figure 5:**
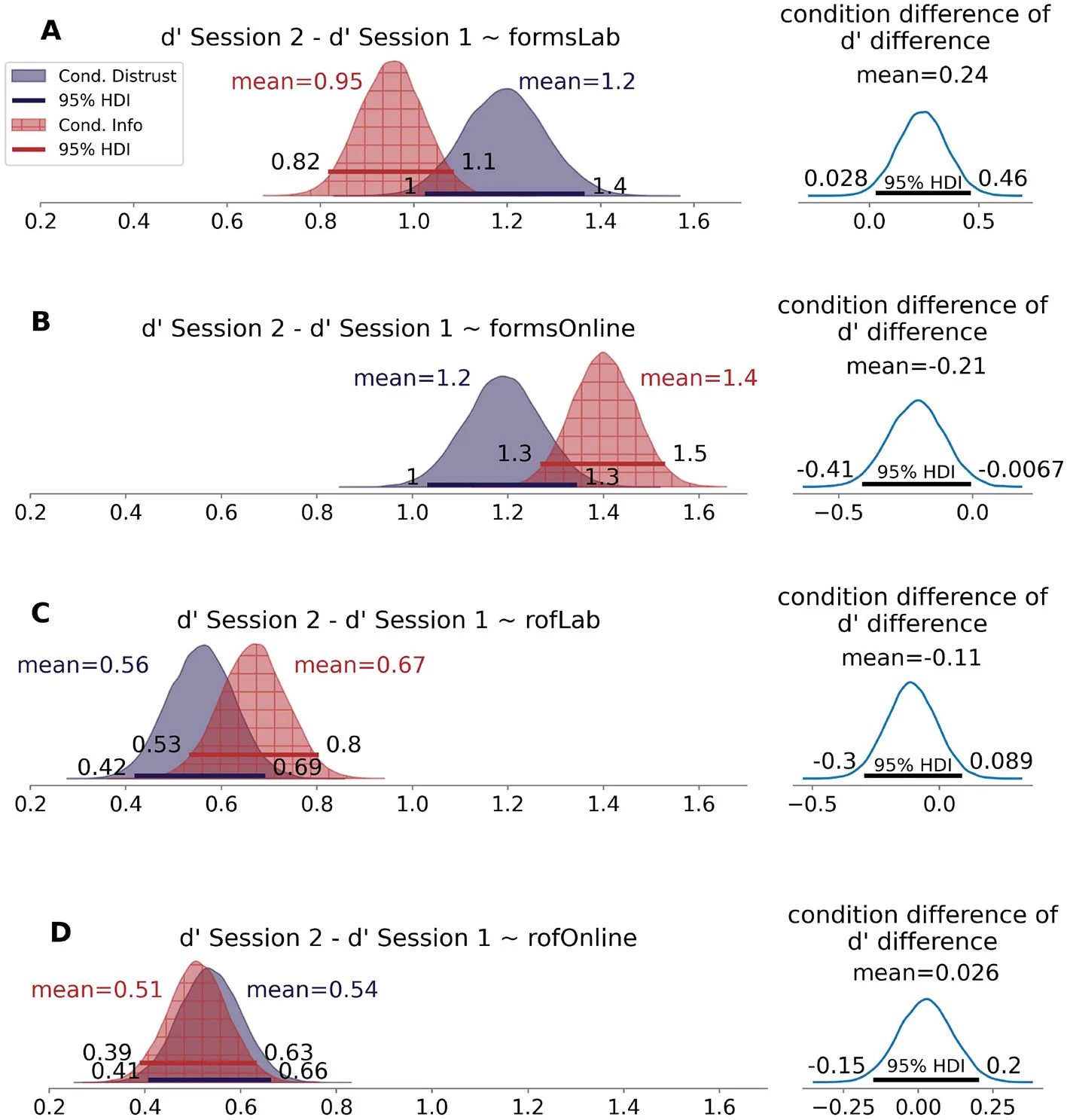
Posterior density distributions of the *d*′ differences of each sub-study (**A**: Forms laboratory, **B**: Forms online, **C**: RoF laboratory, **D**: RoF online).

Visualizing these differences for each study (Figure 5) shows that the expected result pattern occurs only for the laboratory version of the Forms scenario. In the online version of the same scenario, a reverse pattern is present, while a strong overlap between the conditions is observed in the RoF scenario in general. The acceptance rates and switch percentages across all studies also did not differ between conditions (*BF*_10_ = 0.07, *BF*_10_ = 0.03; see Table 3).

**Table 3:** Contingency table for the acceptance rate and switch percentages split by Condition.

**Condition**	**Advice accepted**		**Switched to advice**	
	**No**	**Yes**	**No**	**Yes**
Distrust	3, 052 (23.16%)	10, 124 (76.84)	3, 199 (60.69%)	2, 072 (39.31%)
Info	3, 361 (22.23%)	11, 759 (77.77%)	3, 753 (61.03%)	2, 396 (38.97%)
Total	6, 413 (22.66%)	21, 883 (77.34%)	6, 952 (60.87%)	4, 468 (39.12%)

Difference by Condition for acceptance rate: *BF*_10_ = 0.07.

Difference by Condition for switch percentages: *BF*_10_ = 0.03.

**Figure 6:**
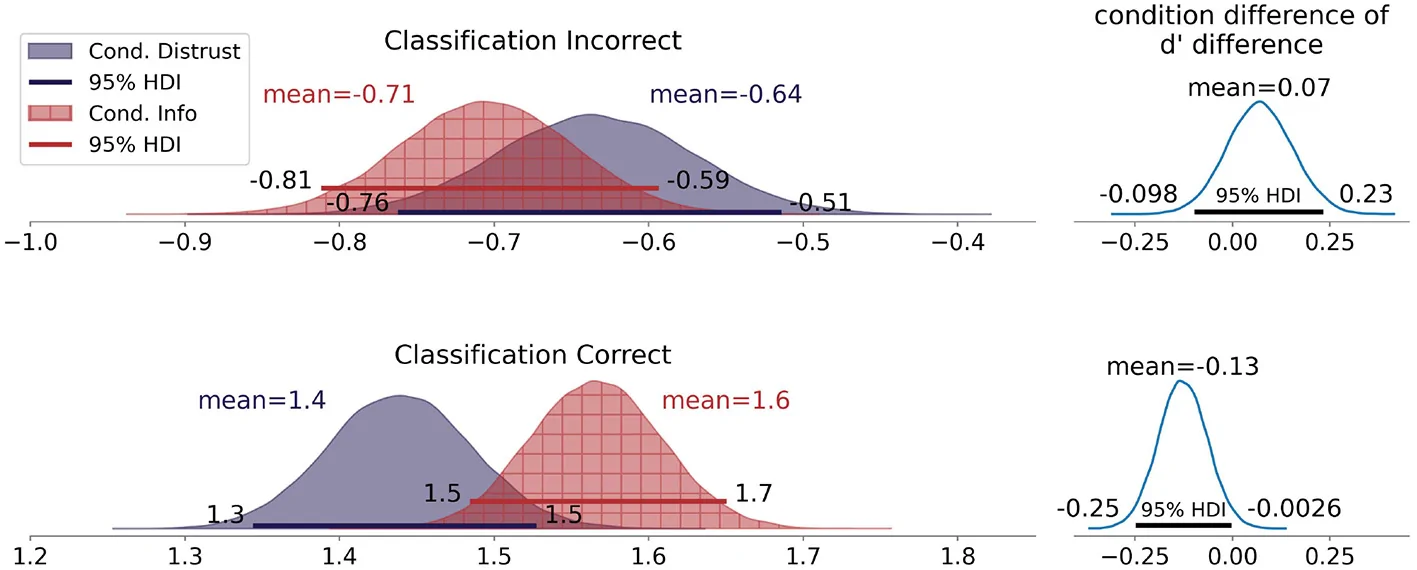
Posterior density distributions of the *d*′ differences between Session 2 and Session 1 split by Condition and Advice Correctness.

Next, we analyzed the detailed *d*′ differences. As Figure 6 shows, when the advice was incorrect, the *d*′ values in the information-only condition (*M* = −0.68, [−0.79, 0.57]) overlap with the *d*′ values in the distrust condition (*M* = −0.64, [−0.76, −0.52]). When considering the data from the trials in which the advice was correct, the *d*′ values in the information-only condition (*M* = 1.5, [1.5, 1.6]) are higher than the values of the distrust condition (*M* = 1.4, [1.3, 1.5]). Looking at the difference of posteriors for the two conditions (Figure 6 right side), we can see that 0 falls within the 95% highest density interval (HDI) for the incorrect advice values and centers at 0.07. Thus, only a tendency of reduced worsening due to incorrect advice in the distrust condition in comparison to the information-only condition is observed. For the trials in which the advice was correct, we see that the 95% HDI of the condition difference does not include 0 with a mean value of –0.13. This indicates a smaller improvement by correct advice in the distrust condition compared to the information-only condition. To interpret this in terms of reliance, the detailed d' differences indicate at most a small reduction of over-reliance in the distrust condition, while this came at the cost of under-reliance in comparison to the information-only condition. In Table 4, we report that the acceptance rates were lower when the advice was incorrect than when it was correct, with no difference between conditions. For the switch percentages, we observed the same pattern (see Table 4). This shows that the participants relied less on incorrect advice than on correct advice, which did not differ between conditions.

**Table 4:** Contingency table for the acceptance rate and switch percentages split by Advice Correctness and Condition.

**Advice correctness**	**Condition**	**Advice accepted**		**Switched to advice**	
		**No**	**Yes**	**No**	**Yes**
Incorrect	Distrust	1, 490 (55.76%)	1, 182 (44.24%)	1, 181 (65.72%)	616 (34.28%)
	Info	1, 657 (53.97%)	1, 413 (46.03%)	1, 329 (65.21%)	709 (34.79%)
Correct	Distrust	1, 562 (14.87%)	8, 942 (85.13%)	891 (25.65%)	2, 583 (74.35%)
	Info	1, 704 (14.14%)	10, 346 (85.86%)	1, 067 (25.96%)	3, 044 74.05%)

Difference by Condition for acceptance rate: *BF*_10*incorrect*_ = 0.08, *BF*_10*correct*_ = 0.04.

Difference by Condition for switch percentages: *BF*_10*incorrect*_ = 0.04, *BF*_10*correct*_ = 0.03.

### 3.2 Self-report

**Figure 7:**
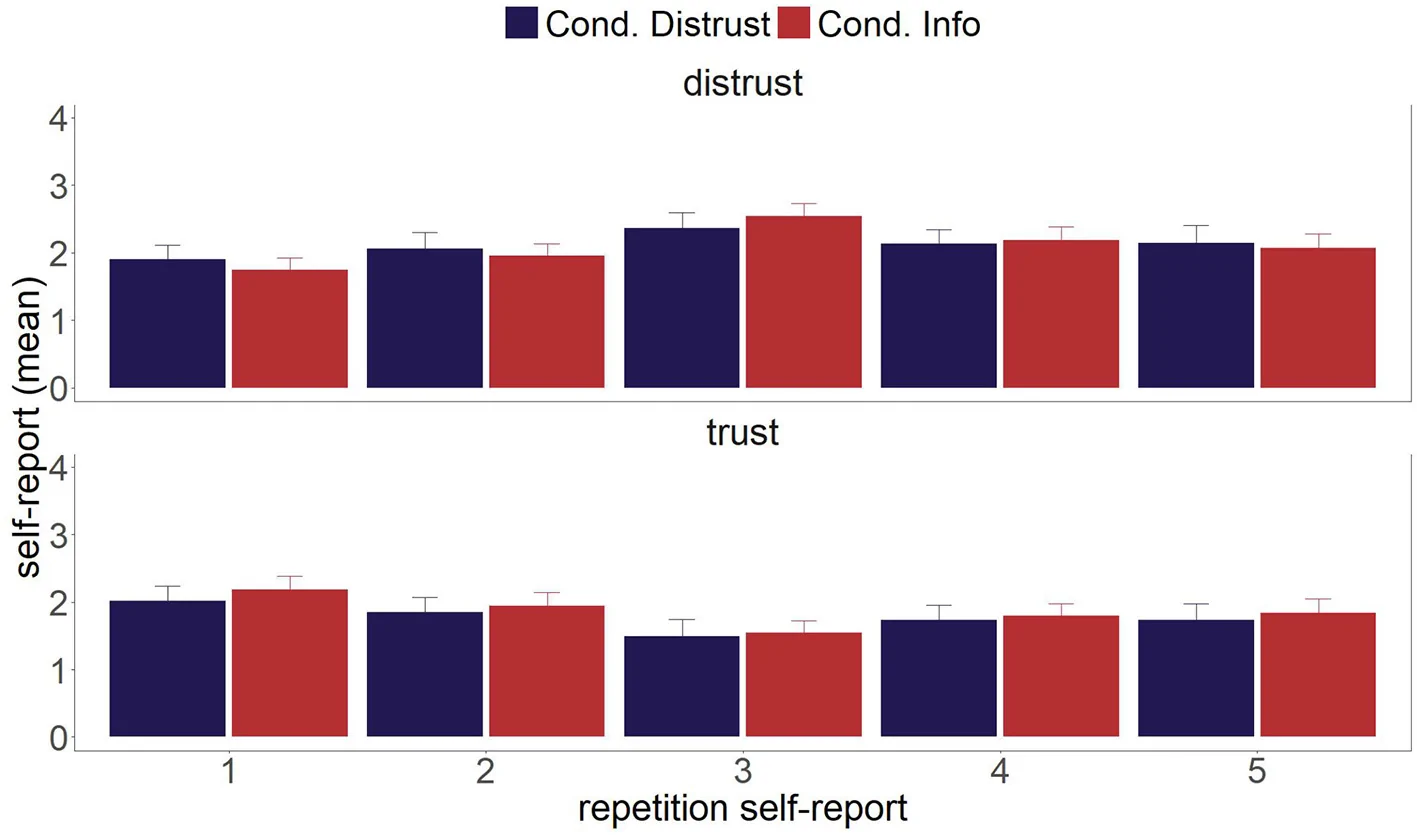
Mean values of self-reported trust and distrust at the five time points during the experiments.

Figure 7 visualizes the mean values of the self-reported trust and distrust for each of the five-time points. To analyze the repeated self-report, we used a Bayesian mixed regression with Condition and the time points of the self-report (queryCount) as fixed effects and the variable participant as a random intercept (see Equation 3). For the variable queryCount we used planned contrast to compare the self-report measurements across the different time points. Their specifications (see Table 5) allow for a group-wise comparison of the time points of the self-report.


(3) $$\begin{array}{l} selfreport\sim Condition*queryCount+\left(1|Participant\right) \end{array}$$

**Table 5:** Contrasts used for the Bayesian mixed regression.

	**Weights**				
Contrast 1	–1	1	0	0	0
Contrast 2	–1	–1	2	0	0
Contrast 3	0	0	2	–1	–1
Contrast 4	0	0	0	1	–1

Figure 7 shows the mean values of the self-reported trust and distrust, each split by Condition. In Tables 6 and 7, we report the results of the mixed regressions with contrasts.^6^ For the predictor Condition, we report the values from the analysis without applying the contrasts to the predictor queryCount. The remaining rows consist of the values from the analysis with the contrasts. A positive estimate value of the predictor Condition indicates higher mean values in the information-only condition than in the distrust condition across all time points.

**Table 6:** Regression coefficients of the Bayesian mixed regression for the self-reported trust.

	**Estimate**	**Est. Error**	**l-95% CI**	**u-95% CI**
Intercept	1.77	0.09	1.59	1.94
Condition	0.17	0.15	–0.12	0.46
queryCount - contrast 1	0.08	0.05	–0.02	0.18
queryCount - contrast 2	0.17	0.04	0.09	0.24
queryCount - contrast 3	0.03	0.04	–0.05	0.10
queryCount - contrast 4	0.00	0.05	–0.10	0.10
Condition:contrast1	0.04	0.07	–0.09	0.17
Condition:contrast2	0.03	0.05	–0.07	0.14
Condition:contrast3	0.01	0.05	–0.09	0.11
Condition:contrast4	–0.02	0.07	–0.15	0.11

l-95% CI = lower boundary of the 95% credible interval, u-95% CI = upper boundary of the 95% credible interval.

**Table 7:** Regression coefficients of the Bayesian mixed regression for the self-reported distrust.

	**Estimate**	**Est. Error**	**l-95% CI**	**u-95% CI**
Intercept	2.12	0.09	1.94	2.30
Condition	–0.16	0.15	–0.46	0.14
queryCount - contrast 1	–0.08	0.05	–0.18	0.01
queryCount - contrast 2	–0.14	0.04	–0.21	–0.06
queryCount - contrast 3	–0.02	0.04	–0.09	0.06
queryCount - contrast 4	–0.01	0.05	–0.10	0.09
ConditionInfo:contrast1	–0.03	0.07	–0.16	0.11
ConditionInfo:contrast2	–0.11	0.05	–0.21	–0.01
ConditionInfo:contrast3	–0.01	0.05	–0.11	0.09
ConditionInfo:contrast4	0.07	0.07	–0.07	0.20

l-95% CI = lower boundary of the 95% credible interval, u-95% CI = upper boundary of the 95% credible interval.

For the self-reported trust, only the 95% credible interval (CI) of Contrast 2 does not include zero. The estimate for this contrast is 0.17[0.08, 0.25], indicating that at time point 3, lower trust was reported than before, thus supporting *Hypothesis 2a*. The other contrasts and their interaction with Condition are close to zero, while their 95% CI includes zero (see Table 6).

For the self-reported distrust (Table 7), a similar but reversed pattern is observed. Again, for Contrast 2, the 95% CI does not include 0, with an estimated value of −0.14[−0.21, −0.06], indicating higher self-reported distrust at time point 3 compared to time points 1 and 2, which supports *Hypothesis 2b*. Furthermore, the interaction between Contrast 2 and Condition, with an estimate of −0.11[−0.21, −0.01], suggests that this increase in distrust was more pronounced in the information-only condition.

Furthermore, Table 8 presents correlations between the mean self-reported trust and distrust and the *d*′ estimates from Session 2, the detailed *d*′ differences, and the acceptance rates and switch percentages are reported. From that, we can see that higher trust goes along with more positive detailed *d*′ differences when the advice was correct and more negative detailed *d*′ differences when the advice was incorrect. Distrust is correlated in the reverse pattern to the detailed *d*′ differences. Both the acceptance rates and the switch percentages are positively correlated with trust and negatively correlated with distrust (see Table 8).

**Table 8:** Correlations between the self-report measures and the *d*′ values from Session 2, including the detailed d' differences, acceptance rate, and switch percentages.

	**Trust**		**Distrust**	
	**r**	**BF_10_**	**r**	**BF_10_**
*d*′ Session 2	–0.21	1.92	0.17	0.70
*d*′ difference - advice correct	0.38	2,195.49	–0.26	9.33
*d*′ difference - advice incorrect	–0.39	3,899.03	0.32	104.56
Acceptance rate	0.37	1,261.06	–0.26	8.11
Switch percentage	0.43	>10,000	–0.25	7.31

*r*= Pearson correlation, *BF*_10_= Bayes factor.

## 4 Discussion

The purpose of this study was to investigate whether distrust is helpful for interaction with erroneous AI. Accordingly, the present experiments examined whether the instruction to remain skeptical and check each AI advice had a positive effect on the combined human-AI performance when interacting with fallible AI. To that end, we compared the *d*′ difference of the participants in a distrust condition to the *d*′ difference of the participants in an information-only condition. Overall, the distrust instruction led to no change in performance, with a small tendency to worsen performance instead of improving it. Out of four experiments, only one indicated a benefit of the distrust instruction. The other three experiments show no effect (RoF - online) or even a negative tendency (RoF - lab and Forms - online).

The reasons for these differing effects of the instruction manipulation are unclear. A clear pattern of results, e.g., similar effects for one type of material or for the laboratory or online setting, is not present. We expected neither a large nor a very robust effect because it was only the initial instruction that was manipulated, but the results were still contrary to our expectations regarding the benefit of the distrust instruction. Instead of the expected benefit, these results illustrate that the effect of instructing to remain skeptical and check each piece of advice may fluctuate or not be present at all. Therefore, the present results call the usefulness of this or similar procedures into question.

Importantly, a parallel exists in the usage of disclaimers in current LLM Chat-Bot applications. Typically, in this context, a disclaimer is presented (Anderl et al., 2024; Bo et al., 2024) either before submitting a prompt or within the chat interface. Such disclaimers resemble our distrust instructions, as they indicate that the generated output may be false or contain wrong information, and should therefore be checked or approached with caution. The present results indicate that such disclaimers may not be effective in shaping how people interact with AI or how they use the generated output. Overall, results regarding distrust instructions are mixed so far. In line with our results, Metzger et al. (2024) reports that a disclaimer about the limitations of an LLM-based conversational agent given prior to the interaction did not alter users' attitudes about the LLM's outputs. However, in another study, disclaimers mitigated over-reliance (Bo et al., 2024). What may be important is that in this study, the disclaimer was presented continuously throughout the interaction, which might be more effective than displaying it only beforehand. Future research should continue to explore this to determine whether repeated disclaimers or a different distrust instruction led to different results than ours. Recently, a qualitative analysis revealed that users' distrust of the generated outputs led to more critical and cautious usage (Colville and Ostern, 2024).

Furthermore, the results of the present study do not indicate the expected improvement in the participants' performance in the post-error phase compared to the pre-error phase. The performances in both phases are very similar, which indicates that the error phase did not alter the participants' usage of AI advice and that they used the advice because the performance in the pre- and post-error phases was higher than the unadvised performance in Session 1. Thus, on average, the participants improved due to the advice, while the increased exposure to wrong advice did not alter this. However, the self-reported trust and distrust indicate that they noticed the worsening of AI advice during the error phase. Participants' distrust increased while their trust decreased because of the error phase. This aligns with a typical pattern where people lose trust upon noticing errors, a phenomenon observed in the context of algorithm aversion (Madhavan and Wiegmann, 2007; Jussupow et al., 2020).

Drawing from the algorithm aversion literature, one might expect a reduction in participants' reliance on advice after the error phase. However, the data indicate no difference in performance between the pre- and post-error phases, suggesting that advice usage remained stable. It is possible that the occasional errors, which occurred during the pre-error phase, may have diminished the impact of the error phase. To explore this possibility more systematically, future studies could ensure that an even higher-quality AI advice is provided in the pre-error phase. Additionally, aligning with recent work on algorithm aversion (Dietvorst et al., 2015; Reis et al., 2024), it would be valuable to label the advice as either human- or AI-generated, or to allow participants to choose which type of advisor to follow. Both approaches could yield further insights into research on algorithm aversion and appreciation (Logg et al., 2019).

Interestingly, the contrast analysis of the self-report reveals that the first drop in AI advice quality (from 90% to 75% correctness) does not lead to a substantial change in the self-report. Only after a further drop in quality (60% and then 45%) is a significant difference in trust and distrust observed. This suggests that either prolonged exposure to errors, a certain frequency of errors, or both need to be present for distrust to arise and for trust to decline. One reason for this may be that with an increased frequency of errors, the errors occurred more continuously. Wang et al. (2024) show that continuous errors are more detrimental to trust than when the same number of errors occur non-continuously.

In addition, we did not observe any substantial changes between the self-reported trust and distrust in the post-error phase and the error phase. For self-reported trust, this aligns with trust research in both interpersonal and human-AI contexts. In multiple studies across different settings (e.g., Lewandowsky et al., 2000; Hoff and Bashir, 2015; Slovic, 1993), it was observed that trust decreases more quickly when expectations are not met (e.g., by noticing errors) than it takes to (re-)gain trust. This is referred to as the asymmetry principle (Poortinga and Pidgeon, 2004; Slovic, 1993), which can be summarized as follows: trust is hard to gain but easy to lose, while the opposite appears to apply to distrust (Vaske, 2016; Guo et al., 2017).

In accordance with previous research that highlights the importance of distinguishing between self-reported trust and the behavioral component of reliance (e.g., Papenmeier et al., 2019, 2022; Wang and Yin, 2023), our results also highlight the differing dynamics of the self-report measures and reliance behavior. While the combined performance in the post-error phase returns to the performance level of the pre-error phase, we did not observe a similar recovery for self-reported trust and distrust. In our study, this discrepancy is also evident in the control variable of self-reported reliance, as this measure returns to the initial level in the post-error phase.

Regarding the conceptualization of trust and distrust as two related yet separate dimensions, our results neither support nor contradict this concept. Trust and distrust were strongly negatively correlated; and correspondingly, trust was negatively correlated with the *d*′ values of Session 2, while distrust was positively correlated with those values. One possible reason for this is that each concept was assessed by only a single item and that participants may have reverse-scored one of them (see below). In accordance with Scharowski and Perrig (2023), we continue to advocate that, based on the underlying theory and existing evidence, the two-dimensional approach is more appropriate. Additional research on trust and distrust, as well as their antecedents and consequences, is necessary to empirically corroborate this.

Conducting two sessions enabled us to control for individual performance differences. The SDT estimates from the two sessions, combined with the correctness of the advice, allowed us to assess whether under-reliance and over-reliance were present and influenced by the manipulated instructions. Contrary to our expectations, we observed only a slight reduction in over-reliance in the distrust condition compared to the information-only condition, while this appeared to result in a rise in under-reliance.

### 4.1 Limitations

The degree to which we can interpret our self-report outcomes is limited. By measuring trust and distrust with only one item each, we have reduced these complex concepts to single statements, which increases the likelihood of overlooking their more detailed aspects. Moreover, the simplicity of this assessment may have led participants to reverse-score the distrust item in relation to the trust item. The single-item measurement of trust and distrust was chosen because we wanted to assess self-reports multiple times during the experiment. We decided in favor of single items and against using a questionnaire due to feasibility. This trade-off between the unobtrusiveness and extensiveness of self-report measurements cannot be easily resolved. Instead, depending on a study's focus, one should be prioritized over the other; however, both are generally very important. Trust and distrust are dynamic, which is why multiple measurements are beneficial. However, their complexity cannot be distilled into single items. Therefore, we consider a more extensive evaluation of self-reported trust and distrust through standardized and validated questionnaires as a promising avenue for future research.

Additionally, although we created some risk for our participants via a monetary incentive, the stakes involved in their behavior were considerably lower compared to the typically high-stakes scenarios found in contexts such as law, finance, or medicine. Thus, our results may not be directly applicable to these contexts. Furthermore, it was not ensured or directly tested whether the participants could accurately recognize the quality of the advice. While the self-report measures indicate that the participants noticed a drop in advice quality, we did not investigate their assessment of the advice directly. For instance, in a study by Miller et al. (2023), participants systematically mistook AI-generated faces for real images due to their higher averageness and familiarity with AI-generated images. Our two-session approach accounted for individual performance differences arising from such potential issues; however, we cannot assert that participants developed true expertise in identifying correct or incorrect advice. To quantify this expertise, future work using a similar approach should involve a direct assessment of the quality of advice, such as measuring the perceived accuracy of AI. Being an expert in the subject at hand may be a key factor for distrust to be beneficial or for having distrust at the correct time in the first place. Thus, applying a similar procedure in different domains with domain experts would be fruitful, and comparable efforts can already be found (e.g., Morrison et al., 2024; Leichtmann et al., 2023).

### 4.2 Conclusion

We argued that trust in AI research may benefit from considering and investigating not only (appropriate) trust but also distrust. We conducted a study consisting of four sub-experiments to investigate whether distrust is helpful when interacting with erroneous image classifications of a mock-up AI. Furthermore, we investigated if a worsening of this AI advice is noticed and how this affected self-reported trust and distrust. Despite the limitations outlined above, this paper makes three contributions.

First, contrary to our expectations, we show that fostering distrust toward erroneous AI advice was not beneficial for the advised performance in our mock-up scenarios. As discussed above, this has implications for such instructions, such as the way disclaimers are currently used in LLM-based chat applications. Second, we provide evidence that people notice declining AI advice and that this affects their self-reported trust and distrust. A decrease in the quality of advice was followed by a drop in trust and a rise in distrust, which did not revert to their initial levels after the advice quality improved. Our data suggest that these shifts in trust and distrust require either prolonged exposure or a higher frequency of errors.

Third, by employing Signal Detection Theory, we were able to analyze the participants' responses without any potential response biases. By conducting two sessions—one with and one without AI advice—with a known ground truth, we contribute an analysis that assesses reliance in detail to evaluate whether over- or under-reliance occurs and to compare the extent to which they are mitigated. The unadvised Session 1 controls for individual performance differences in classifying the material. Simultaneously, the differences in the *d*′ estimates of the two sessions quantify potential improvements or deteriorations due to AI advice. In addition to investigating between-subject factors such as the present condition manipulation, this approach can also be applied to within-subject manipulations; for example, to compare different classification accuracies, designs, or the addition of various XAI methods.

Future research on appropriate reliance should adopt approaches like ours that allow for the investigation of mitigating under- and over-reliance. By fully studying this underlying aim of appropriate trust and related notions, their understanding would progress. Despite the wording of these notions and the focus on evaluating trust in this context, we advocate that distrust should also be considered. Otherwise, one would evaluate only the presence or absence of trust; while, due to the possibility of errors, not the absence of trust, but the presence of distrust can be warranted.

## Figures and Tables

**Figure A1:**
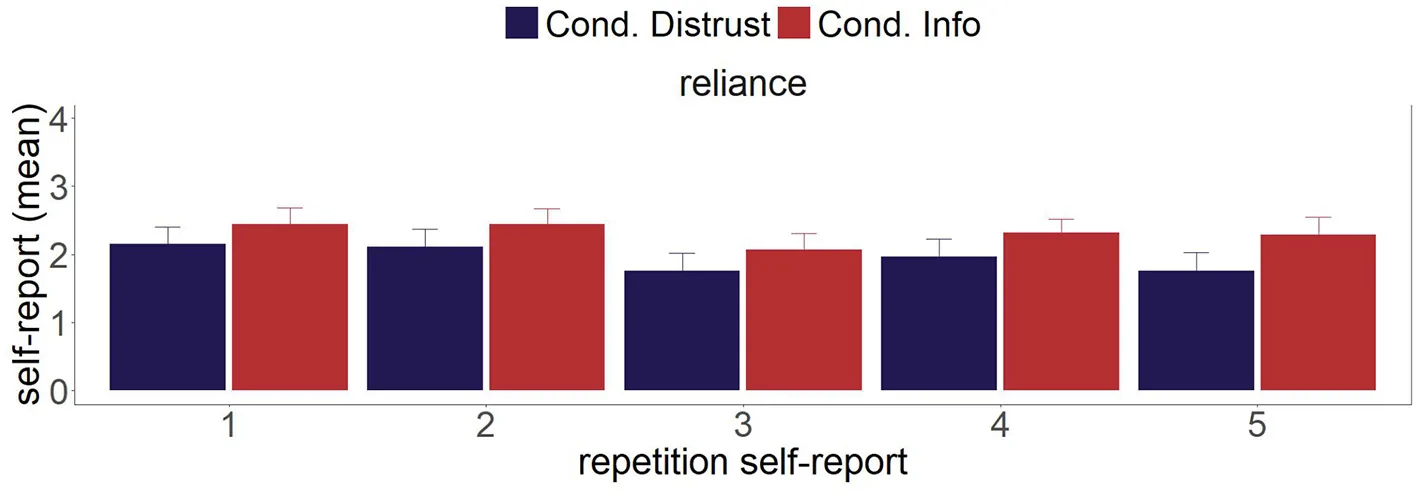
Mean values of the control variable self-reported reliance at the five time points during the experiments.

## Data Availability

The datasets presented in this study can be found in online repositories. The names of the repository/repositories and accession number(s) can be found below: OSF repository: https://osf.io/y3hgk/?view_only=22755d69b3cf4cbd8bb8bd1072f64342.

## Footnotes

1 The full code is provided on GitHub; (see Data availability statement).

2
*https://www.prolific.com/*

3 All stimuli and details on the material generation and selection can be found on GitHub; see available data (Data availability statement).

4 For more information on the generation, see *whichfaceisreal.com* and Karras et al. (2019).

5 The script can be found on GitHub; (Data availability statement).

6 In the Appendix, we also visualize the self-reported reliance that was assessed as a control variable and report the results of the mixed regression for this measure.

## Appendix

### Additional results for the control variable self-reported reliance

Table A1 reports the results of the Bayesian mixed regression for the control variable self-reported reliance. The 95% CI of Contrast 2, with an estimate of 0.18[0.10, 0.26], does not include 0, indicating a decrease at time point 3 compared to time points 1 and 2. Furthermore, the estimates of Contrast 3 (0.09[0.01, 0.16]) and Contrast 4 (0.11[0.01, 0.21]) are positive, indicating an increase in self-reported reliance when comparing time points 3 to 4 and 5, as well as when comparing time points 4 and 5.

**Table A1:** Regression coefficients of the Bayesian mixed regression for the self-reported reliance.

	**Estimate**	**Est. Error**	**l-95% CI**	**u-95% CI**
Intercept	1.95	0.11	1.73	2.17
Condition	0.29	0.18	–0.06	0.63
queryCount - contrast 1	0.02	0.05	–0.08	0.12
queryCount - contrast 2	0.18	0.04	0.10	0.26
queryCount - contrast 3	0.09	0.04	0.01	0.16
queryCount - contrast 4	0.11	0.05	0.01	0.21
Condition:contrast1	–0.02	0.07	–0.15	0.12
Condition:contrast2	–0.05	0.05	–0.16	0.05
Condition:contrast3	–0.07	0.05	–0.18	0.03
Condition:contrast4	–0.09	0.07	–0.23	0.04

l-95% CI = lower boundary of the 95% credible interval, u-95% CI = upper boundary of the 95% credible interval.
