# Supplementary material for: Interacting with fallible AI: is distrust helpful when receiving AI misclassifications?
Source: Front Psychol. 2025 May 27;16:1574809. doi: 10.3389/fpsyg.2025.1574809 (PMC12149757; doi:10.3389/fpsyg.2025.1574809)
Supplement: Supplementary file 1 [file Table_1.docx]

**Forms**

**Instruction Information Only**

In the following, you will receive AI support. The AI trained to classify the shapes has already been successfully evaluated. As a preliminary study showed that the test subjects had difficulties recognising small and relevant differences in the height-to-width ratio of the shapes, the AI was specially trained for this.

This can still lead to occasional misclassifications. Overall, however, the AI performs very well.

You should therefore use the AI classifications to help you make your decision!

-- german original --

Im Folgenden erhalten Sie nun KI-Unterstützung. Die auf die Klassifikation der Formen trainierte KI wurde bereits erfolgreich evaluiert. Da eine Vorstudie zeigte, dass die Probanden Schwierigkeiten hatten, kleinere und relevante Unterschiede im Höhen-Breiten-Verhältnis der Formen zu erkennen, wurde die KI speziell hierauf trainiert.

Dadurch kann es immer noch gelegentlich zu Fehlklassifikationen kommen. Insgesamt weist die KI aber eine sehr gute Performanz auf.
Nutzen Sie daher die KI-Klassifikationen als Hilfe bei Ihrer Entscheidung!

**Instruction Distrust**

In the following, you will receive AI support. The AI trained to classify the shapes has already been successfully evaluated. As a preliminary study showed that the test subjects had difficulties recognising small and relevant differences in the height-to-width ratio of the shapes, the AI was specifically trained for this.

Overall, the AI performed very well. However, the focus on training the AI can still lead to occasional misclassifications. These errors can sometimes be very obvious to humans. Even for less obvious cases, previous studies have shown that humans often have good intuition and can recognise when a classification is incorrect by critically examining it.

You should therefore use the AI classifications to help you make your decision but always check for yourself whether the AI classifications seem sensible to you.

-- german original --

Im Folgenden erhalten Sie nun KI-Unterstützung. Die auf die Klassifikation der Formen trainierte KI wurde bereits erfolgreich evaluiert. Da eine Vorstudie zeigte, dass die Probanden Schwierigkeiten hatten, kleinere und relevante Unterschiede im Höhen-Breiten-Verhältnis der Formen zu erkennen, wurde die <b>KI speziell hierauf trainiert.

Insgesamt weist die KI eine sehr gute Performanz auf. Durch den Fokus beim Training der KI kann es aber immer noch gelegentlich zu Fehlklassifikationen kommen. Diese Fehler können für Menschen teilweise sehr offensichtlich sein.    Auch für weniger offensichtliche Fälle haben bisherige Untersuchungen gezeigt, dass Menschen oft eine gute Intuition haben und durch kritisches Prüfen gut erkennen können, wann eine falsche Klassifikation vorliegt.
Nutzen Sie daher die KI-Klassifikationen als Hilfe bei Ihrer Entscheidung, aber prüfen Sie stets für sich, ob Ihnen die KI-Klassifikationen sinnvoll erscheinen.

**RoF**

**Instruction Information Only**

In the following, you will receive AI support. The AI trained to classify the images has already been successfully evaluated.

There may still be occasional misclassifications, but overall the AI performs very well.

You should therefore use the AI classifications to help you make your decision!

-- german original --

Im Folgenden erhalten Sie nun KI-Unterstützung. Die auf die Klassifikation der Bilder trainierte KI wurde bereits erfolgreich evaluiert.

Es kann zwar immer noch gelegentlich zu Fehlklassifikationen kommen, aber insgesamt weist die KI eine sehr gute Performanz auf.
Nutzen Sie daher die KI-Klassifikationen als Hilfe bei Ihrer Entscheidung!

**Instruction Distrust**

In the following, you will receive AI support. The AI trained to classify the images has already been successfully evaluated.

Overall, the AI performs very well. However, misclassifications can still occur occasionally. These errors can sometimes be very obvious to humans. Even for less obvious cases, previous studies have shown that humans often have good intuition and can recognise when a classification is incorrect by critically examining the data.

You should therefore use the AI classifications to help you make your decision but always check for yourself whether the AI classifications seem sensible to you.

-- german original --

Im Folgenden erhalten Sie nun KI-Unterstützung. Die auf die Klassifikation der Bilder trainierte KI wurde bereits erfolgreich evaluiert.

Insgesamt weist die KI eine sehr gute Performanz auf. Es kann aber immer noch gelegentlich zu Fehlklassifikationen kommen. Diese Fehler können für Menschen teilweise sehr offensichtlich sein. Auch für weniger offensichtliche Fälle haben bisherige Untersuchungen gezeigt, dass Menschen oft eine gute Intuition haben und durch kritisches Prüfen gut erkennen können, wann eine falsche Klassifikation vorliegt.
Nutzen Sie daher die KI-Klassifikationen als Hilfe bei Ihrer Entscheidung, aber prüfen Sie stets für sich, ob Ihnen die KI-Klassifikationen sinnvoll erscheinen.
